# Evaluation of Buccal Bone Thickness and Root Length of Maxillary Incisors in Deep Bite Patients Using Cone Beam Computed Tomography

**DOI:** 10.1002/cre2.70207

**Published:** 2025-08-28

**Authors:** Zahra Alsadat Emami Meybodi, Omid Mortezai, Zeynab Shalli, Maryam Tofangchiha, Elham Emami Meybodi, Abolfazl Razi Avarzamani

**Affiliations:** ^1^ Department of Orthodontics, Dental Caries Prevention Research Center Qazvin University of Medical Sciences Qazvin Iran; ^2^ Department of Oral and Maxillofacial Radiology, Dental Caries Prevention Research Center Qazvin University of Medical Sciences Qazvin Iran; ^3^ Oral and Maxillofacial Radiologist Yazd Iran; ^4^ Qazvin University of Medical Sciences Qazvin Iran; ^5^ Oral and Maxillofacial Surgery Resident, Craniomaxillofacial Research Center, Oral and Maxillofacial Surgery Department, School of Dentistry Tehran University of Medical Sciences Tehran Iran

**Keywords:** CBCT, deep bite, incisor

## Abstract

**Objectives:**

The aim of this study is to evaluate the buccal bone thickness and root length of the maxillary incisors in deep bite patients.

**Material and Methods:**

Cone beam computed tomography data of 124 patients were randomly selected in this cross‐sectional study. In the study, patients were divided into two groups, normal bite and deep bite, and buccal bone thickness and root length of the maxillary incisors were measured. Buccal bone thickness was measured at 4 mm apical to the cemento–enamel junction and at the midpoint of the root.

**Results:**

By comparing these factors between the two groups of normal and deep bite, only the difference in the buccal bone thickness at the midpoint of the root of the central incisor between these two groups was statistically significant. Also, by comparing these factors between male and female patients, the root length of the central and lateral incisors and the buccal bone thickness in the mid root of the central incisor were significantly more in Men.

**Conclusion:**

A significant disparity in the buccal bone thickness at the mid‐root level of central incisors between normal bite and deep overbite patients was revealed.

**Abbreviations**
BBT: buccal bone thickness
CBCT: cone beam computed tomography
CEJ: cemento–enamel junction
CEJ‐4: 4 mm apical to cemento–enamel junction
mm: millimeter
MR: midpoint of the root

## 1 Introduction

Deep bite is a common occlusal characteristic that is seen in many dental and skeletal malocclusions (Cenzato et al. 2021). The thickness of the alveolar bone in the anterior region of the jaw is one of the important factors in determining the possible range of tooth movement in orthodontic treatment. An important factor when placing mini screws for orthodontic purposes is bone thickness (Al Amri et al. 2020). Computed tomography is the most reliable method to measure the root length of the tooth (Al Amri et al. 2020; Baumgaertel et al. 2017).

The craniofacial morphology is a complex system with phenotypic variations determined by genetics, environmental factors, and functional demands. The growth of the maxillomandibular region is related to the surrounding muscular system. The craniofacial structure, including oral soft tissue and hard tissue, are influenced by the strain of the masticatory force (Weinmann 1955; Ichim et al. 2007).

Vertical facial morphology is important for the orthodontist, influencing growth prediction, anchorage system, bite force and function. Since the vertical facial dimensions are related to morphological bone change controlled by genetics and function during childhood (Bresin 2001), it is reasonable to believe that maxillary and mandibular cortical bone thickness might differ in patients with different facial growth patterns (Tsunori et al. 1998; Chan et al. 2008; Gaffuri et al. 2021).

Considering the high demands of patients for orthodontic treatment and also the necessity of special consideration during orthodontic treatment of teeth with low Anchorage value and with different buccal bone thickness, the aim of this study is to evaluate the buccal bone thickness and root length of the maxillary incisors in deep bite patients.

## 2 Methods and Materials

In this cross‐sectional descriptive‐analytical study, cone beam computed tomography (CBCT) data of 136 patients from two offices in Karaj and Tehran were selected. Based on the results of a similar study witch Shrestha R et al. assessed correlation of anterior overbite with root position and buccal bone thickness of maxillary anterior teeth, and considering the maximum standard deviation of bone thickness as the main outcome of the study 1.12, type I error of 0.005 (*Z* = 0.961), power of study 0.8 and a precision of study 0.2, a minimum of 121 samples was required. The sampling method in this study was convenience sampling. The data was confidential, and a numerical code was used to record the data. Before starting the study, the research objectives were explained to the patients, and informed consent was obtained from them for the use of radiography. This study was conducted with the code of ethics IR.QUMS. REC.1402.095 in Qazvin Dental Faculty. The inclusion criteria were fully erupted permanent teeth (with or without third molar), no history of orthodontic treatment, periodontitis, temporomandibular joint disease, lip and palate cleft or other abnormalities of the skull and face, Jaw cyst or tumor, facial trauma and orthognathic surgery and anterior tooth crowding not more than 4 mm (Li et al. 2022). After examining the patients, the CBCT data of 124 patients who defined inclusion criteria were selected. The devices used in the offices to produce CBCT were KODAK 8100 and Planmeca, with the exposure conditions of 90 volts KVP and variable time and current based on patient's age and size. The Field of View (FOV) with which the acquisitions were made in CBCT was small. These CBCT scans were related to patients aged 18–40.

Patients in the study were divided into two categories, normal bite and deep bite based on overbite over than 4 mm and between 0 and 4 mm, also buccal bone thickness and incisor root length were measured. Due to measuring the length of the root in the longitudinal axis of the tooth and measuring the thickness of the buccal bone in the axiolabiolingual plane of each tooth, which are independent from the maxillofacial planes, there is no interference in terms of standardization of the investigated plane.

To measure the length of the incisor root, the longitudinal axis of the tooth from the incisal to the apex in the axiolabiolingual plane of the tooth had been drawn and the distance from the cemento–enamel junction (CEJ) to the apex of the tooth had been measured by two investigators, an arothodontics resident and an oral and maxillofacial radiologist. Buccal Bone Thickness (BBT) also was measured at 4 mm apical to the CEJ (CEJ‐4) and at the midpoint of the root (MR).

Buccal bone thickness and root length were compared between two groups and also between male and female patients. Data analysis was done using SPSS version 25 software, and an independent *t*‐test was used to investigate the relationship between buccal bone thickness and root length with normal or deep bite variables. The significance level of the test was considered as *p* < 0.05.

This data sets used and/or analysed during the current study are available from the corresponding author on reasonable request.

## 3 Results

Out of 124 samples, 48 (38.7%) were men and 28 (22.6%) of the examined patients were deep bite.

The average root length of the maxillary central and lateral incisors respectively were 13.25 ± 1.59 and 13.42 ± 1.51 mm, the thickness of the buccal bone at the midpoint of the central and lateral incisor roots respectively were 0.83 ± 0.26 and 0.84 ± 0.40 mm, and the buccal bone thickness at the CEJ‐4 of central and lateral incisors were 0.84 ± 0.30 and 0.87 ± 0.40 mm, respectively.

As shown in Table 1, There was no statistically significant difference between the two groups with normal bite and deep bite (*p* > 0.05) in root length of the maxillary central and lateral incisors, the buccal bone thickness of the maxillary lateral incisors at the mid root and the maxillary buccal bone thickness at CEJ‐4 point in the central and lateral incisors. While there was a statistically significant difference (*p* = 0.009) between the two groups in the thickness of the buccal bone of maxillary central incisors at the midpoint of the root.

**Table 1:** Comparison of the variables based on the type of the overbite.

	Bite type	Sample size	Average	Standard deviation	*p*‐value (independent *t*‐test)
Root Length of maxillary central teeth	Normal	94	13.20	1.64	0.570
	Deep	27	13.40	1.41	
Root Length of maxillary lateral teeth	Normal	94	13.41	1.44	0.862
	Deep	28	13.47	1.77	
BBT of maxillary central teeth at MR	Normal	94	0.86	0.26	0.009
	Deep	27	0.71	0.25	
BBT of maxillary lateral teeth at MR	Normal	94	0.85	0.36	0.513
	Deep	28	0.78	0.53	
BBT of maxillary central teeth at CEJ‐4	Normal	94	0.84	0.30	0.761
	Deep	27	0.82	0.28	
BBT of maxillary lateral teeth at CEJ‐4	Normal	94	0.84	0.38	0.186
	Deep	28	0.96	0.48	
Root Length of maxillary incisors	Normal	96	13.29	1.43	0.701
	Deep	28	13.41	1.51	
BBT of maxillary incisors at MR	Normal	96	0.86	0.27	0.274
	Deep	28	0.77	0.39	
BBT of maxillary incisors at CEJ‐4	Normal	96	0.84	0.30	0.291
	Deep	28	0.92	0.40	

Intraclass correlation coefficients (ICC) value for root length assessing of the teeth was 0.95 ± 0.09. It was 0.88 ± 0.15 for the buccal bone thickness at CEJ‐4 of the incisors and 0.85 ± 0.12 for the buccal bone thickness at midpoint of the incisors.

There was a statistically significant difference between male and female patients in the root length of the maxillary central and lateral incisors (*p* = 0.001) and the thickness of the buccal bone at the midpoint of the root of the incisors and central maxillary incisors (*p* = 0.006) (Table 2).

**Table 2:** Comparison of the variables according to gender.

	Gender	Sample size	Average	Standard deviation	*p*‐value (Independent *t*‐test)
Root Length of maxillary central teeth	Male	48	13.81	1.53	0.001
	Female	73	12.88	1.52	
Root Length of maxillary lateral teeth	Male	48	13.98	1.15	0.001
	Female	74	13.06	1.61	
BBT of maxillary central teeth at MR	Male	48	0.91	0.24	0.006
	Female	73	0.77	0.26	
BBT of maxillary lateral teeth at MR	Male	48	0.90	0.36	0.147
	Female	74	0.79	0.42	
BBT of maxillary central teeth at CEJ‐4	Male	48	0.85	0.32	0.725
	Female	73	0.83	0.28	
BBT of maxillary lateral teeth at CEJ‐4	Male	48	0.90	0.39	0.554
	Female	74	0.85	0.42	
Root Length of maxillary incisors	Male	48	13.89	1.17	< 0.001
	Female	76	12.95	1.49	
BBT of maxillary incisors at MR	Male	48	0.90	0.26	0.048
	Female	76	0.79	0.32	
BBT of maxillary incisors at CEJ‐4	Male	48	0.88	0.29	0.693
	Female	76	0.85	0.35	

## 4 Discussion

Since the assessment of the amount of labial bone covering the maxillary incisors is needed in diagnostic purpose and treatment plan (Gracco et al. 2008; Baysal et al. 2013; Raber et al. 2019; Jia et al. 2015; Sendyk et al. 2017), the present study was conducted to investigate the thickness of the buccal bone and the root length of the maxillary incisors in deep bite patients by using CBCT.

Anteroposterior movement of anterior teeth often occurs during orthodontic treatment, and excessive movement can lead to unpleasant complications such as root resorption, dehiscence, or fenestration (Raber et al. 2019).

In a review study conducted in 2018, the prevalence of deep bite was reported as 21.98% (Alhammadi et al. 2018), which is very close to the results of our study.

The prevalence reported in the present study is different from Proffitt et al.'s report (Bhateja et al. 2016; Proffit et al. 2019) which stated that the prevalence of deep bite is about 20% in children and about 13% in the adult population, which can indicate the high prevalence of this malocclusion in our target society.

The average root length of maxillary central and lateral incisors was 13.25 ± 1.59 and 13.42 ± 1.51 mm, respectively. The thickness of the buccal bone at the midpoint of the central and lateral incisor roots was 0.83 ± 0.26 and 0.84 ± 0.40 mm, respectively, and the buccal bone thickness at the CEJ‐4 of central and lateral incisors was 0.84 ± 0.30 and 0.88 ± 0.40 mm, respectively. In a similar study, the average buccal bone thickness at CEJ‐4 was reported as 0.89 ± 0.51 mm for central incisors and 0.85 ± 0.68 mm for lateral incisors (Shrestha et al. 2019), which is close to the results of our study. In the study conducted on Chinese patients, the thickness of the buccal bone at the midpoint of the root for the central and lateral incisors was reported as 0.77 ± 0.44 mm and 0.59 ± 0.55 mm, respectively (Shrestha et al. 2019), which is less than the results obtained in the present study.

In a similar study (Shrestha et al. 2019), there was a positive relationship between overbite and Buccal bone thickness in CEJ‐4. Also, in the study of Gaffuri et al. (2021) in 2021, the thickness of the buccal apical bone and the thickness of the mid‐root bone on the palatal side showed statistically significant differences in hyperdivergent versus hypodivergent individuals, which shows that the cortical bone is thicker in the hypodivergent group, although in the current study, the cortical bone thickness of the palatal region was not investigated.

In the study conducted by Li et al. (2022), unlike the present study, no correlation was found between the vertical indices of the face and the patient's overbite with the thickness of the buccal bone of the upper incisors. In a study that investigated and compared the root length in patients with normal bite and open bite, no significant difference was reported in terms of root length of maxillary incisors between these two groups (Arriola‐Guillén et al. 2020). In this study, we also did not observe a significant difference in the root length of these teeth between the two groups.

In the study conducted by Uehara et al. (2013), which examined the length of the root of the teeth between the open bite and normal bite groups, the shorter length of the teeth in the open bite group was observed due to the loss of occlusal contact; since the occlusal contact in deep bite patients is established in the anterior region, the results of the present study can be expected.

In a study conducted in 2017 (Capitaneanu et al. 2017), tooth length variables had the greatest potential for gender recognition. A study conducted in Latin America in 2023 (Yang et al. 2023) reported a significant difference in tooth size between men and women, in such a way that men showed larger tooth crown dimensions than women, which is similar to the results of the present study.

The indicators of the present study revealed that the thickness of the buccal bone at the midpoint of the root of the maxillary central incisors (*p* = 0.006) is significantly greater in men than in women. In a study (Hassan and Khazaal Al‐Jaboori 2022) that examined the thickness of the buccal bone of the central tooth in three points, the crest, the midpoint of the root and the apex of the root, as well as the thickness of the alveolar bone, there was sexual difference in the thickness of the buccal bone, as in the present study. Men have stronger chewing muscles than women to apply biting force, so that the presence of greater thickness of the labial bone covering the incisor teeth is quite logical.

The results of our study show that in male patients, none of the studied indicators had a statistically significant difference between the normal bite and deep bite groups, but in female patients, the buccal bone thickness at the midpoint of the central incisor root in normal bite patients was significantly greater than deep bite patients. The unequal number of female and male samples and the higher number of females in the above study could be a reason for the similarity of the results in females and the total population.

Considering that during orthodontic treatment, deep bite patients suffer more root resorption in central and lateral teeth compared to normal bite and open bite patients (Salehi et al. 2009), The results of this study raises concerns about the increased risk of root resorption during orthodontic intervention in deep bite patients which potentially reduces our ability to use temporary anchorage devices in the maxillary anterior region of deep bite patients. The use of appropriate technique and biomechanics in deep bite patients, according to the results of the present study, is much more important, and we are obliged to treat by mechanics that prevent the application of heavy force in deep bite patients.

This study also emphasizes the importance of sexual differences in dental anatomy, which men showing longer root lengths and thicker buccal bone in the mid‐root region of central incisors. These differences require a gender‐specific approach to orthodontic treatment planning; According to the findings, we probably need therapeutic mechanics with a higher force than what is used in female patients. Considering the sexual difference in the amount of buccal bone in the incisor region of the maxilla at the midpoint of the upper central root, the possibility of using temporary anchor devices in the anterior region of maxilla of men increases compared to women, although this issue requires more studies focused on the use of Bone anchors in this area of alveolar bone.

As a limitation of the study, the resolution and the voxel matrix could influence the precision of the measurement with two decimal digits.

In general, considering that some studies have shown that CBCT has a relatively lower positive predictive value in the examination of thin bone (Sun et al. 2015), it is impossible to comment definitively on the results of our study, and accurate judgment requires conducting similar studies. Also, it can be beneficial to conduct investigations on root length before and after treatment in deep bite patients with different mechanics of orthodontic treatment. Also including adolescents and open bite patients in the studies in future can be beneficial.

## 5 Conclusion

A significant difference in the buccal bone thickness at the mid‐root level of central incisors between normal bite and deep bite patients was revealed. Additionally, significant gender‐based difference was observed, with men displaying greater root length in both central and lateral maxillary incisors and a thicker buccal bone, suggesting the influence of gender on dental and skeletal characteristics in deep overbite condition. These findings highlight the necessity of orthodontic treatment planning which considers individual anatomical variations to enhance treatment efficacy.

## Author Contributions

Zahra Alsadat Emami Meybodi collected data and analyzed data, and wrote the manuscript. Omid Mortezaii, Zeinab Shali, and Maryam Tofangchiha checked and helped in writing the manuscript and collecting data. Elham Emami Meybodi collected and analyzed data. Abolfazl Razi Avarzamani helped in writing the manuscript and submitting the data.

## Conflicts of Interest

The authors declare no conflicts of interest.

## Data Availability

Data openly available in a public repository that issues data sets with DOIs.
